# Chinese Herbal Medicine Compound Microecological Agent (C-MEA) Improves Egg Production Performance in Caged Laying Ducks via Microbiota–Gut–Ovary Axis

**DOI:** 10.3390/vetsci12090808

**Published:** 2025-08-25

**Authors:** Yanfeng Lu, Lei Zhang, Rui Zhu, Xiujun Duan, Guobo Sun, Yuying Jiang

**Affiliations:** 1 School of Animal Science and Technology, Jiangsu Agri-Animal Husbandry Vocational College, Taizhou 225300, China; luyanfeng@jsnmkjzyxy6.wecom.work (Y.L.); leizhang@jsahvc.edu.cn (L.Z.); rzhu2021@jsahvc.edu.cn (R.Z.); sgb1981@126.com (X.D.); m19517822335@163.com (Y.J.); 2 Jiangsu Key Laboratory for High-Tech Research and Development of Veterinary Biopharmaceuticals, Engineering Technology Research Center for Modern Animal Science and Novel Veterinary Pharmaceutic Development, Taizhou 225300, China

**Keywords:** *Intestinal microbiota*, caged laying ducks, egg production, C-MEA, follicle development

## Abstract

This study was conducted to investigate the effects of a Chinese herbal medicine compound microecological agent (C-MEA) on the egg production performance, ovarian follicle development, ovary transcriptome, and cecal microbiota of caged laying ducks. A total of 108 black Muscovy ducks (150 days old) were randomly divided into three groups for 30 days in a formal feeding trial. Compared with the control basic diet (Group C) and 16 g/kg C-MEA dosage (Group B), the 8 g/kg C-MEA dosage (Group A) increased egg production (average laying rate 69.35%) and follicle development (5~7 Fs, 6~7 LYFs, 11~13 SYFs) mass (*p* < 0.05). According to RNA-Seq, the ovaries’ transcriptome among different dietary groups enriched six key pathways, including neuroactive ligand–receptor interaction, the PPAR signaling pathway, ECM–receptor interaction, focal adhesion, the adherens junction, and the FoxO signaling pathway, as well as 46 candidate key genes. According to 16S-Seq, the microbial diversity was significantly increased in Group A, and the genus abundances of *Sphaerochaeta* and *UCG-004* were significantly changed among different dietary groups (*p* < 0.05). Supplementation with C-MEA may optimize the cecal microflora and the interactions between the intestinal microflora and the host. The results from combining RNA-Seq and 16S-Seq demonstrated that the relationship between *Sphaerochaeta* and the hub gene cluster (*F2*, *KNG1*, *C5*, *PLG*, *F2RL1*, *FABP1*, and *GCG*) is the most prominent. In conclusion, the egg performance of caged laying ducks can be modulated through the microbiota–gut–ovary axis. Our findings provide new insights for improving gut health and reproductive performance of caged laying ducks.

## Simple Summary

Farming laying ducks and processing their eggs is a traditional industry in China that is gradually transitioning towards modern cage-rearing systems to achieve large-scale and high-efficiency production. However, cage rearing alters ducks’ natural behaviors, making them prone to individual stress, which may lead to pathological conditions, ultimately impacting laying performance. To explore antibiotic-free additives, this study investigated the effects of a Chinese herbal medicine compound microecological agent (C-MEA) on the egg production performance, ovarian follicle development, ovary transcriptome, and cecal microbiota of caged laying ducks. The results show increased egg production and follicle development due to up- and downregulated candidate key genes, as well as increased cecal microbiota diversity. Our findings suggest that the C-MEA enhances egg production, ovary function, and microbial diversity, offering new insights into improving the gut health and reproductive performance of caged laying ducks.

## 1. Introduction

The farming of laying ducks and duck egg processing are a traditional industry in China, accounting for over 90% of global laying duck production [1]. In 2024, the laying duck population reached approximately 163 million in stock, yielding 2.914 million tons of commercial duck eggs, with a total industry value of CNY 41.0 billion [2]. With the continuous expansion of breeding scale and improvements in standardized raising levels, traditional water- or ground-based rearing systems can no longer meet the needs of industrial development. There is an urgent need to transform duck farming methods to alleviate environmental pressure and further enhance productivity [3]. Compared to conventional free-range or captive breeding systems, cage-rearing systems for laying ducks enable intensive, large-scale production through mechanization with high breeding efficiency. This approach significantly improves farming efficiency while facilitating the centralized management of manure and waste, thereby substantially reducing environmental pollution [4]. However, cage rearing alters ducks’ natural behaviors, making them prone to individual stress, which may lead to diarrhea, compromised immunity, increased mortality, and altered microbial diversity, thereby ultimately impacting laying performance [5,6,7]. Consequently, implementing effective measures to mitigate stress in caged laying ducks holds critical significance.

With the current comprehensive ban on antibiotics in the raising industry and the rising concern over antibiotic resistance, the search for non-toxic, residue-free antibiotic alternatives has become an urgent need. Microecological agents, a novel class of biological additives designed to rebuild and optimize the diversity of animal gut microbiota, may promote nutrient absorption, enhance intestinal barrier function, and modulate the host’s immune response [8,9]. Traditional Chinese herbal medicine, derived from natural animals and plants, exhibits antibacterial, anti-inflammatory, stress-reducing, and endocrine-regulating effects, which can significantly improve the growth performance, immune function, and gut microbiota composition of animals. To combine both advantages of microecological agents and traditional Chinese herbal medicine, a specific novel microecologic agent (C-MEA) has gradually become a research hotspot in animal husbandry [10]. Wang et al. [11] compared Chinese herbs, probiotics, and prebiotics with antibiotics on the performance of Pekin ducks, and they demonstrated that the additives of Chinese herbs and probiotics had no side effects on the ducks. Gao et al. [12] reported that combining traditional Chinese medicine with probiotics in broiler chicken feed created a synergistic effect, improving the growth performance, immune function, and intestinal health of broiler chickens. Compared with antibiotics, C-MEA offers significant advantages, including safety, environmental compatibility, residue-free properties, and the absence of drug resistance, and it shows significant value for potential application as a feed additive within animal husbandry. However, the application of C-MEA in animal husbandry production remains in the exploratory stage, with particularly limited research reports on its application in the duck rearing industry.

In this study, three dietary inclusion levels of C-MEA were designed, and the regulatory effects on laying performance, ovarian follicle development, ovary transcriptome, and cecal microbiota were evaluated in caged laying ducks. The aim of this study was to understand in-depth the changes in production properties, ovary function, and intestinal health of caged laying ducks in response to the inclusion of dietary supplements of different levels of C-MEA in order to furnish a reference basis for implementing C-MEA in the duck industry and to facilitate the transition towards environmentally sustainable animal husbandry.

## 2. Materials and Methods

### 2.1. Preparation of C-MEA

The C-MEA (Q/01JXDD ST05-2021) used in this experiment was provided by JINXIUDADI Animal Pharmaceutical Co., Ltd. (Shijiazhuang, Hebei, China). The main ratio was 20 g of Huangqi, 15 g of licorice, 15 g of Ciwujia, 10 g of Houttuynia cordata, 10 g of dandelion, 10 g of chrysanthemum, 10 g of lily, 10 g of motherwort, as well as 0.15 g of Enterococcus faecalis, 0.1 g of Bacillus subtilis, and 0.1 g of Clostridium butyricum.

### 2.2. Experimental Design and Sample Collection

A total of 108 female 150-day-old black Muscovy ducks individually housed in distinct single cages were raised at the National Gene Bank of Waterfowl Resources in Jiangsu, China. The ducks were randomly divided into 3 groups by a single-factor experimental design (36 ducks per group): (1) Group A, basic diet + 8 g/kg C-MEA; (2) Group B, basic diet + 16 g/Kg C-MEA; and (3) Group C, basic diet without C-MEA. The basic diet was the same commercial formula diet (Twin Biochemical Co., Ltd., Hangzhou, Zhejiang, China), which mainly contained corn, wheat, and soya bean meal. The addition of C-MEA was based on our previous study. The total experimental period was 37 days, including 7 days of the pre-trial period (P1–7) and 30 days of the formal period (F1–30). At the end of the trial period, 6 ducks per group were randomly selected for sampling. Ovaries and digesta of the cecum were carefully collected and promptly snap-frozen in liquid nitrogen for subsequent experiments.

### 2.3. Laying Performance

Eggs were picked up manually at 16:00 every day under the same management conditions. Duck egg production was recorded daily to calculate the daily laying rate. At the end of the experiment, a total of 90 valid individual records were collected.

### 2.4. Analysis of Ovarian Follicles

A total of 18 ovarian tissues were separated and compared as representative individuals among three groups. According to the follicle diameter, the follicles of different grades were divided into preovulatory follicle (F), large yellow follicle (LYF), and small yellow follicle (SYF) categories, as described by Gilbert [13] and Kun Zou et al. [14]. The Fs, LYFs, and SYFs were counted in three sections from each individual.

### 2.5. Ovary Transcriptome Data Analysis

Three ovarian stroma samples were selected randomly from each group, and then the qualified total RNA was extracted and transported to Novogene Co., Ltd. (Beijing, China) for transcriptome sequencing (RNA-Seq). The libraries were sequenced on an Illumina HiSeq 2500 system sequencing platform. The clean reads were mapped to the Muscovy duck’s reference genome (GCA_048319975.1) using hisat2 (2.0.5) for the downstream analysis as previously described [15]. The differential expressed genes (DEGs) between samples were identified using the DESeq2 R package (1.20.0) [16], and *p* < 0.05 and log_2_|foldchange| ≥ 1 were used as the criteria of significance of DEGs. The GO term and KEGG pathway analyses were performed by clusterProfiler software (3.8.1) [17], and *p* < 0.05 was considered significantly enriched. The STRING database [18] was used to explore the interaction between DEGs. The sequencing raw data were deposited into the Genome Sequence Archive in the BIG Data Center (https://ngdc.cncb.ac.cn/gsa, accessed on 3 August 2025) with accession number PRJCA044169.

### 2.6. Quantitative Reverse Transcription Polymerase Chain Reaction (qRT-PCR) Analysis

To validate the RNA-Seq results, the expression of 7 randomly selected DEGs was assessed using qRT-PCR. Total RNA was extracted from ovary samples using the TRIzol Kit (Invitrogen). The primers (Supplementary Table S1) were designed using Primer 5.0 software. qPCR was performed on a CFX96 Real-Time System (Bio-RAD). The relative gene expression levels of selected DEGs were quantified based on *β-actin* gene expression by the 2^−ΔΔCt^ method.

### 2.7. Cecal Microbiota Analysis

Genomic DNA was extracted from cecum contents using the QIAamp 96 PowerFecal QIAcube HT kit (QIAGEN, Germany) according to the manufacturer’s protocols and then evaluated by 1% agarose gels. Then, two DNA samples from each group were pooled as a 16S sequencing sample (*n* = 3 per group, pooled from 6 individuals). The 16S rDNA V3-V4 region of the ribosomal RNA gene was amplified from genomic DNA using forward primer 341F: CCTACGGGNGGCWGCAG, 806R: GGACTACHVGGGTATCTAAT, of which the PCR requirements described in [19] were followed. The PCR product was analyzed on 2% agarose gel electrophoresis, purified with the QIAquick gel extraction kit (QIAGEN, Frankfurt, Germany). Then, the amplicons were subsequently sequenced (16S-Seq) using the Illumina NovaSeq 6000 platform, and bio-informatics analysis was performed by Novogene Co., Ltd. (Beijing, China). The sequencing raw data were deposited into the Genome Sequence Archive in the BIG Data Center (https://ngdc.cncb.ac.cn/gsa) with accession number PRJCA044144.

The raw fastq files were merged by FLASH (1.2.11) to obtain the raw tags, which were then filtered by fastp (0.23.1) to obtain the clean tags. Using the Silva database (https://www.arb-silva.de) and Unite database (https://unite.ut.ee), these tags were compared to obtain the effective tags, which were clustered and classified into operation taxonomic units (OTUs) of ≥95% similarity by utilizing QIIME2 software (202202). The Tax4Fun2 software package (201807) was applied to predict the functional capabilities of microbial communities. The Spearman correlation co-efficient between environmental factors and genera was calculated using the R project psych package (version 1.8.4) [20].

### 2.8. Statistical Analysis

For 16S sequencing data, the alpha and beta diversity analysis was evaluated by QIME2 (202202). The alpha and beta indices were compared across groups with Kruskal–Wallis and ANOSIM tests using R software (V3.4.3), respectively. All statistical analyses in this study were performed with statistical testing using one-way analysis of variance (ANOVA). The results are expressed as mean ± standard deviation. * signifies a significant difference between means (*p* < 0.05), and ** signifies an extremely significant difference between means (*p* < 0.01).

### 2.9. Ethics Statement

The protocol was performed after the approval of the Committee on Experimental Animal Management of Jiangsu Agri-Animal Husbandry Vocational College, Taizhou, China (No. Jsahvc-2023-94), and every effort was made to minimize animal suffering during the experiments.

## 3. Results

### 3.1. Effects of C-MEA on Laying Performance of Caged Laying Ducks

**Figure 1:**
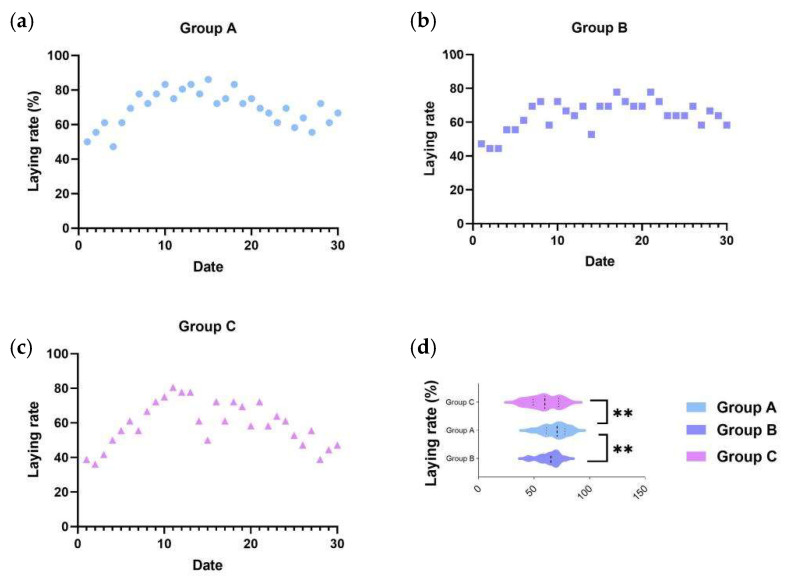
The effects of C-MEA on the laying performance of caged laying ducks, specifically Groups (**a**) A, (**b**) B, and (**c**) C, and laying rate comparasions among three groups (**d**). ** represents an extremely significant difference between means (*p* < 0.01).

To accurately assess the effect of the C-MEA on the egg-laying performance of caged ducks, we collected the 30-day egg production rates of all three groups after a 7-day pre-trial period. Based on the records, an egg production rate ≥ 75% was defined as the peak laying period, as these belongs to the top 25% of total egg production, while egg production rate ≤ 55% (bottom 25%) was defined as the low-laying period. As shown in Figure 1a, Group A (8 g/kg) maintained the peak laying period from E5 to E30 and subsequently declined slowly to approximately 66.67%, of which the average egg production rate was 69.35%; Group B (16 g/kg) maintained peak production from E4 to E30 and subsequently declined slowly to 58.33%, of which the average egg production rate was 65.00%; and Group C (basic diet control) exhibited the lowest egg production efficiency (59.17%), maintained peak production only from E5 to E24, and then dropped to 47.22% at the end of the trial period. From the results, Group A demonstrated a significant improvement in egg production performance (Figure 1d).

### 3.2. Effects of C-MEA on Follicular Development of Caged Laying Ducks

**Figure 2:**
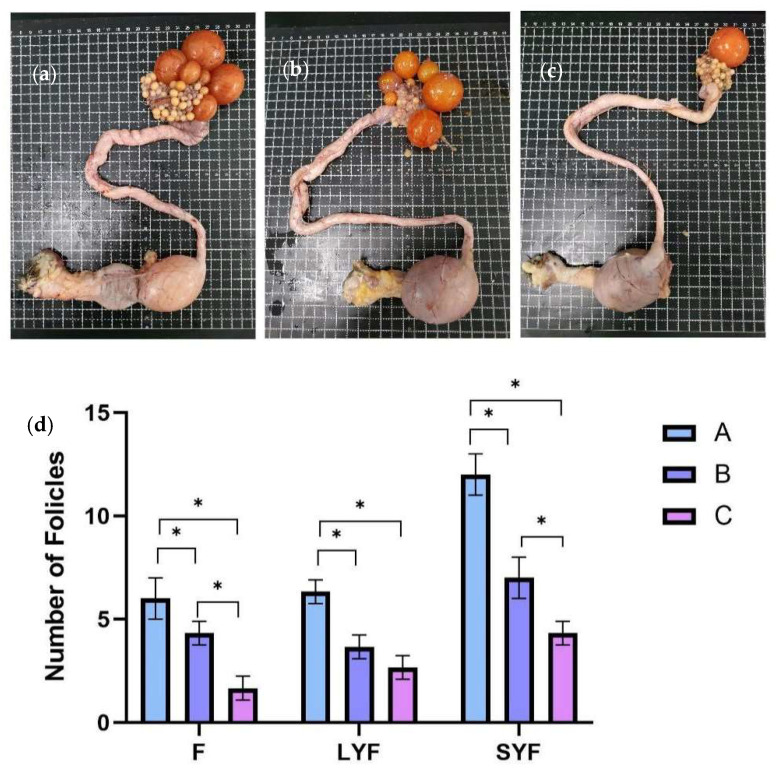
The effects of C-MEA on the follicular development of caged laying ducks, specifically Groups (**a**) A, (**b**) B, and (**c**) C, and the comparasions of follicles’ numbers among three groups (**d**). * represents an extremely significant difference between means (*p* < 0.05).

At E30, F, LYF, and SYF numbers were counted in three groups from 18 individuals (Figure 2a–c). As shown in Figure 2d, there were significantly more Fs, LYFs, and SYFs from Group A than those from Groups B and C. Based on the egg-laying records, the egg-laying efficiency of Group C was lower than 50%, and there were only 1~2 preovulatory follicles on its ovary (Figure 2a). Meanwhile, there were 5~7 and 4~5 preovulatory follicles in Groups A and B, respectively. There were 6~7, 3~4, and 2~3 LYFs in Groups A, B, and C, respectively. There were only 4~5 SYFs in Group C, whereas Group A had 11~13 and Group B had 6~8. The Fs, LYFs, and SYFs in the C-MEA groups (Groups A and B) were all significantly increased compared to those of the control group (Group C) (*p* < 0.05).

### 3.3. Analyzing RNA-Seq Data as Compositions

**Figure 3:**
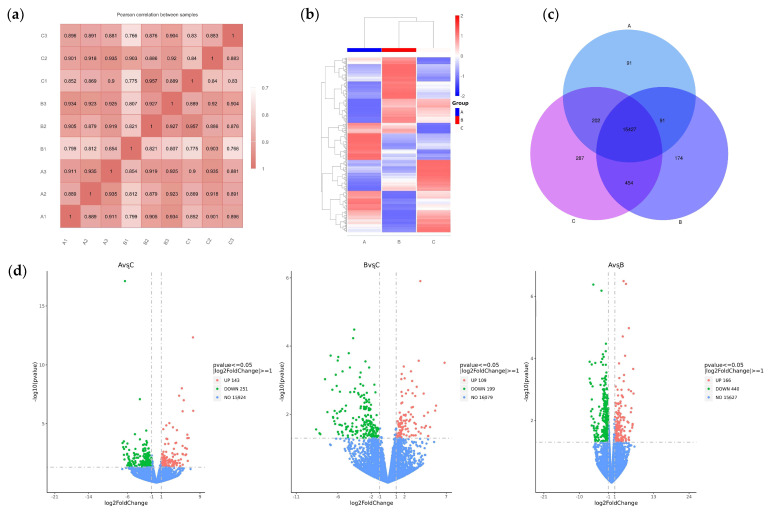
RNA-Seq-based analysis of DEGs in the ovaries of caged laying ducks from different dietary groups. (**a**) The correlation heatmap between samples. (**b**) The cluster analysis result of DEGs in different groups. (**c**) The Venn analysis result of DEGs in different groups. (**d**) The volcano plot of up- and downregulated DEGs of different pairwise comparisons.

To analyze the mechanism by which the Chinese herbal medicine compound microecological preparation improved the egg production performance of caged laying ducks, transcriptome sequencing was performed on the ovaries of ducks in Groups A, B, and C at E30. Through library construction and platform sequencing, the raw data were filtered by quality control, and nine samples were obtained with 43, 482, 166~49, 194, and 710 clean data (see Supplementary Table S2). The proportion of bases with an accuracy rate > 99.9% (Q30) in the total sequence after filtering exceeded 92.8%. This indicates that the transcriptome data collected in this experiment were of high quality and that the sequencing data obtained were reliable, making them suitable for subsequent analysis. Based on the positional information of gene alignments on the reference genome, the FPKM (fragments per kilobase of exon model per million mapped fragments) of all genes in each sample were quantified. Subsequently, correlation coefficients within and between groups were calculated by FPKM values of all genes across samples. These correlations were visualized in a heatmap, as shown in Figure 3a, and the biological replicates within each group clustered together, indicating a distinct sample grouping situation. The DEGs of the three groups were analyzed by DESeq2 (1.20.0) (Figure 3b). Venn analysis showed that a total of 16,726 DEGs were obtained from the transcriptomes of the three ovarian groups, including 91 DEGs specific to Group A, 174 DEGs specific to Group B, and 287 DEGs specific to Group C (Figure 3c). Pairwise comparison showed 143 up- and 251 downregulated DEGs in A vs. C, 109 up- and 199 downregulated DEGs in B vs. C, and 166 up- and 440 downregulated DEGs in A vs. B (Figure 3d).

**Figure 4:**
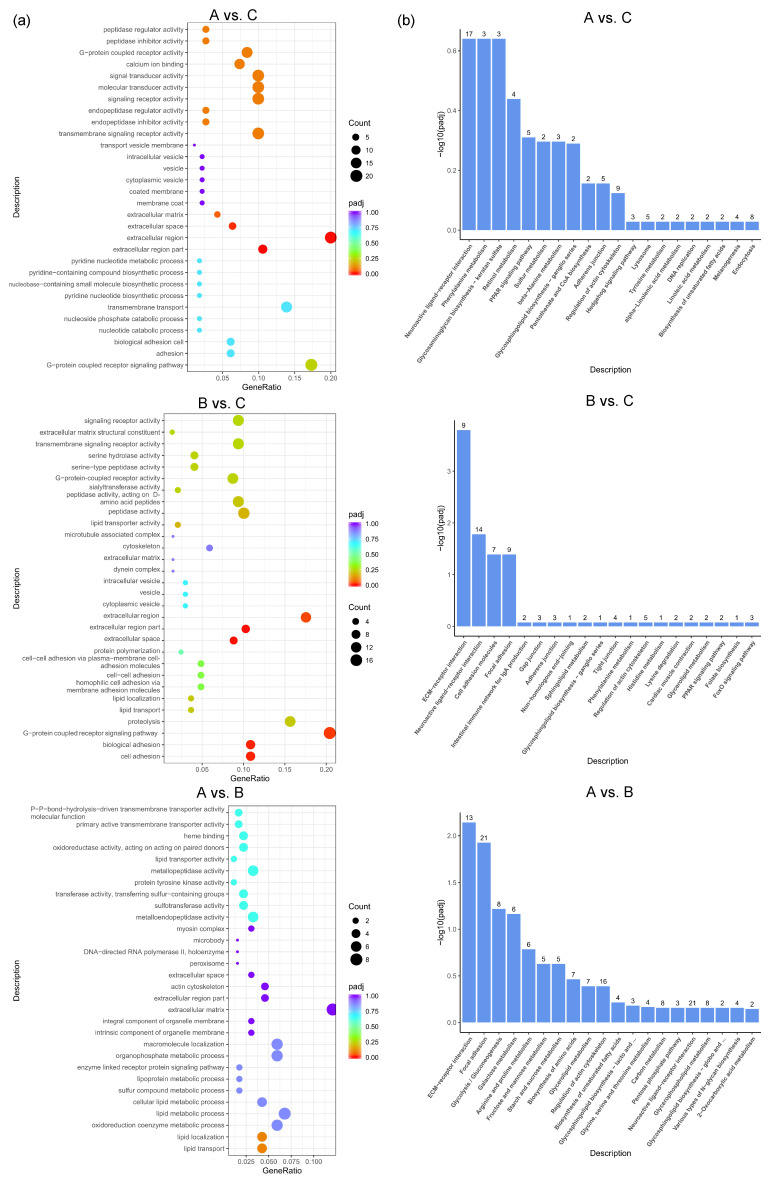
GO and KEGG analysis based on DEGs between different dietary groups of caged laying ducks. (**a**) Top 30 GO classifications and (**b**) top 20 KEGG analysis based on DEGs between different dietary groups of caged laying ducks, the number above the column represents the number of genes enriched in the pathway.

GO enrichment analysis was performed to further express the functional roles of DEGs in different feed groups. The top 30 most significant GO terms in the three pairwise comparisons are shown in Figure 4a. The most significant GO terms related to egg production in A vs. C included the G-protein-coupled receptor signaling pathway, calcium ion binding, extracellular matrix, etc. The most significant GO terms related to egg production in B vs. C included lipid transport, the extracellular region, G-protein-coupled receptor activity, etc. The most significant GO terms related to egg production in A vs. B included the oxidoreduction coenzyme metabolic process, lipid transport, extracellular matrix, etc. To better understand the biological functions and interaction of genes, KEGG pathway analysis was performed for the identified DEGs. The top 20 significantly enriched pathways (*p* < 0.05) by DEGs of each pairwise comparison are shown in Figure 4b, including common reproductive-related pathways such as neuroactive ligand–receptor interaction, the PPAR signaling pathway, beta-alanine metabolism, pantothenate and CoA biosynthesis, the hedgehog signaling pathway, and the lysosome in A vs. C; ECM–receptor interaction, neuroactive ligand–receptor interaction, focal adhesion, the adherens junction, the PPAR signaling pathway, and the FoxO signaling pathway in B vs. C; and ECM–receptor interaction, focal adhesion, and neuroactive ligand–receptor interaction in A vs. B.

**Figure 5:**
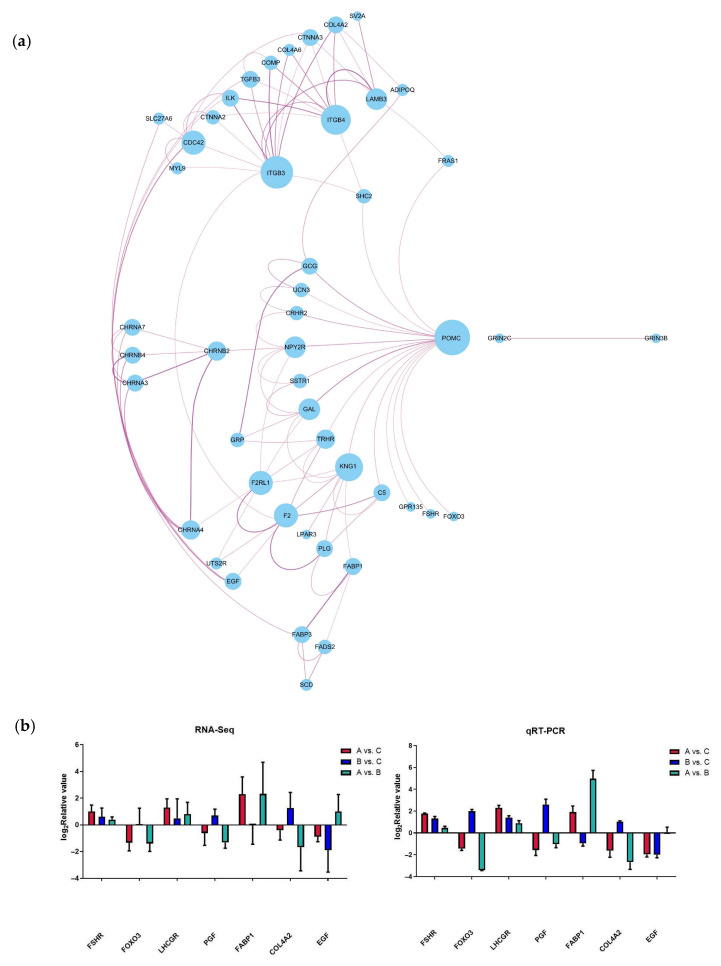
Analysis and verification of candidate key genes in the ovary among different dietary groups of caged laying ducks. (**a**) Interaction network of candidate key genes from RNA-Seq. (**b**) Verification of candidate key genes using qRT-PCR.

Based on the significance levels of KEGG pathways and the literature reviews of the function of these pathways, six potential pathways were considered related to the laying performance of caged ducks associated with Chinese herbal medicine compound microecological preparations, including neuroactive ligand–receptor interaction, the PPAR signaling pathway, ECM–receptor interaction, focal adhesion, the adherens junction, and the FoxO signaling pathway. Therefore, 73 DEGs were enriched in these six signaling pathways and defined as candidate genes potentially mediating the effect of the microecological preparation of Chinese herbal medicine compound on the differences in egg production performance among the different feed groups of caged laying ducks. To investigate the molecular regulatory mechanisms of these 73 DEGs, a network of mRNA interactions was established by Cytoscape software (3.7.2). Two groups of gene interaction clusters are shown in Figure 5a, and the top cluster had 46 DEGs, which were defined as candidate key genes that may regulate egg production based on the addition of C-MEA. Seven candidate key genes were randomly selected for qRT-PCR validation (Figure 5b). A comparison of RNA-Seq and qRT-PCR data showed that these genes exhibited consistent expression trends, which suggested that transcriptomic sequencing data and candidate key genes from RNA-Seq had high reliability and accuracy.

### 3.4. Analyzing 16S rRNA Gene Tag Sequencing Data as Compositions

**Figure 6:**
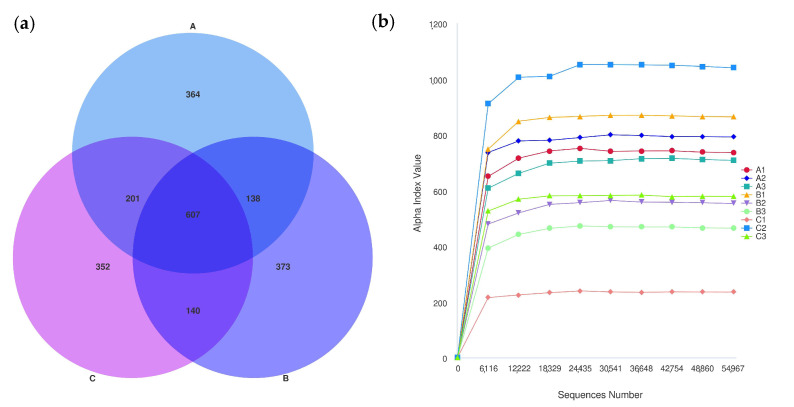
Numbers of cecal OTUs in the three dietary groups. (**a**) The rarefaction curves of Good’s coverage reached saturation in different groups. (**b**) The Venn diagram of OTUs of cecal microbiota in different groups of laying ducks.

**Figure 7:**
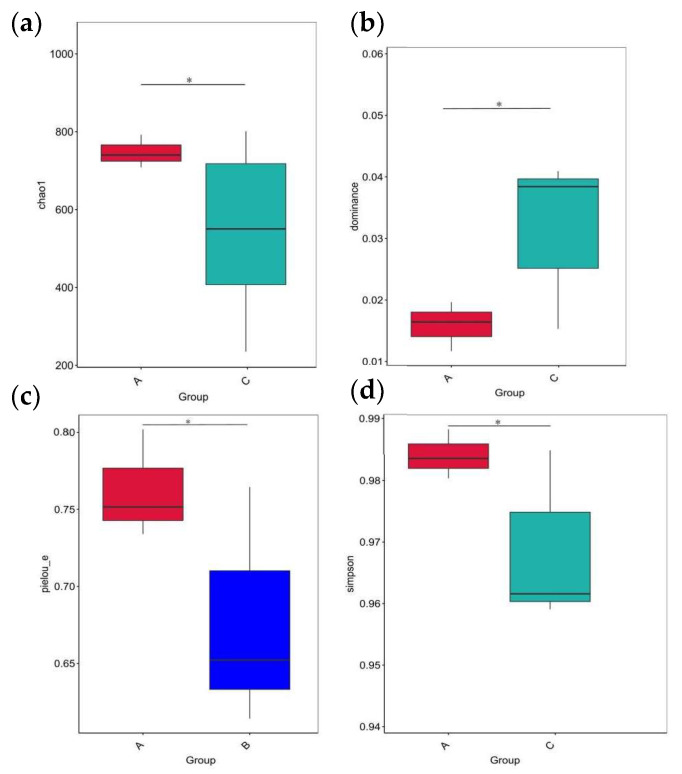
The alpha diversity of cecal microbiota between different dietary groups. (**a**) The Chao1 index of cecal microbiota between Groups A and C. (**b**) The dominance index of cecal microbiota between Groups A and C. (**c**) The Pielou_e index of cecal microbiota between Groups A and B. (**d**) The Simpson index of cecal microbiota between Groups A and C. * indicates statistical significance (*p* < 0.05).

After the initial filtering and adaptor trimming process, samples from caged Muscovy ducks with different feeds accounted for a total of 933,790 RawPE. After sequence assembly, data filtration, and chimera removal, 839,307 effective tags were obtained, which were classified into 818,289 effective tags (Nochime). The number of reads that passed through each step of assembly and control are presented in Supplementary Table S3. Group A displayed the greatest number of OTUs, and the three groups shared 607 OTUs that were common to the cecum, as shown in Figure 6a. The rarefaction curves for the groups were infinitely close to the saturation plateau, proving that the sequencing results contained enough depth to capture most microbial diversity information and could be used for further analysis (Figure 6b). Based on the 2175 OTUs, the alpha diversity of cecal bacterial communities was compared between different dietary groups. A significant difference in the Chao1, dominance, and Simpson indices was observed between the A and C groups (*p* < 0.05, Figure 7a,b,d), and the dietary A group had a significantly higher richness in the Pielou_e index than the B group (*p* < 0.05, Figure 7c).

**Figure 8:**
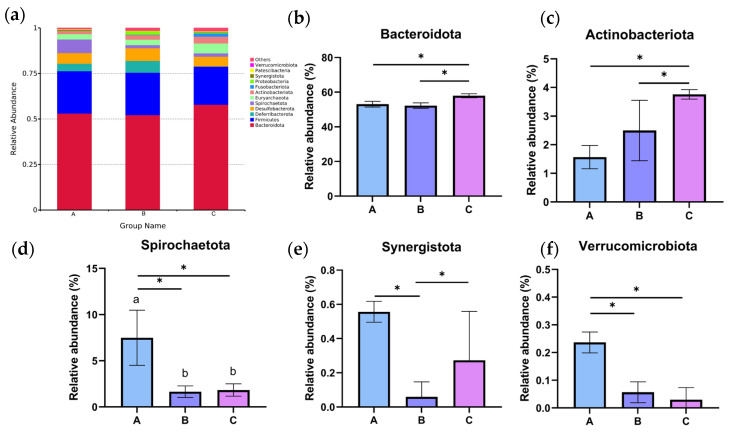
The relative abundance of the cecal microbiota at the phylum level in different dietary groups based on the 16S rDNA gene sequence. (**a**) The stack-column of the cecal microbiota from different groups at the phylum level. (**b**–**f**) The relative abundance of *Bacteroidota*, *Actinobacteriota*, *Spirochaetota*, *Synergistota*, and *Verrucomicrobiota* is expressed as mean ± SEM. * indicates significant differences at *p* < 0.05.

Based on the analysis of the bacterial composition of different dietary groups at the phylum level, *Bacteroidota* and *Firmicutes* dominated all three groups (Figure 8a). Furthermore, the relative abundance of *Spirochaetota*, *Synergistota*, and *Verrucomicrobiota* was clearly higher than those in the C group (*p* < 0.05, Figure 8d–f), while Chinese medicine combined with treatment with composite microecological preparations decreased the relative abundance of *Bacteroidota* and *Actinobacteriota* (*p* < 0.05, Figure 8b,c).

**Figure 9:**
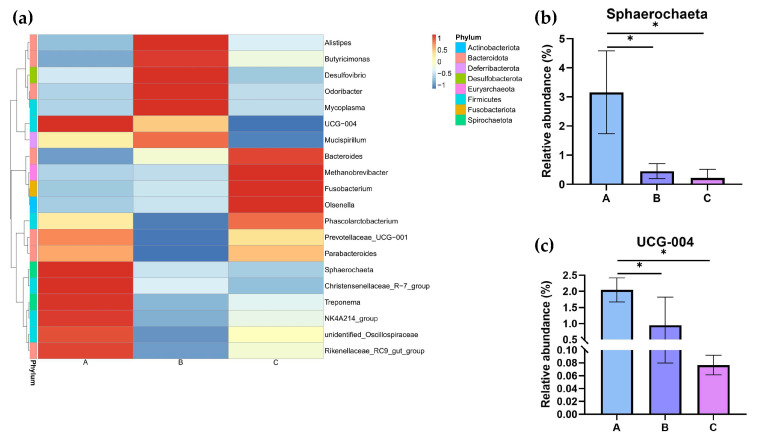
The relative abundance of the cecal microbiota at the genus level in different dietary groups based on the 16S rDNA gene sequence. (**a**) The stack-column of the cecal microbiota from different groups at the genus level. (**b**,**c**) The relative abundance of *Sphaerochaeta* and *UCG-004* is expressed as mean ± SEM. * indicates significant differences at *p* < 0.05.

At the genus level, the top 20 bacterial taxa were clustered among the three groups to form heatmaps (Figure 9a). The relative abundances of *Sphaerochaeta* and *UCG-004* were determined (Figure 9b,c). Compared to Groups B and C, the relative abundances of *Sphaerochaeta* and *UCG-004* were significantly changed in Group A (*p* < 0.05).

**Figure 10:**
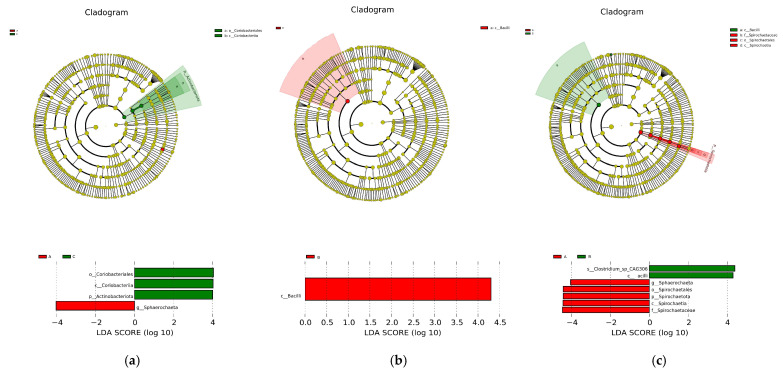
The main taxa of microbiota that were different in the three dietary groups. A cladogram of the main taxa of microbiota that were different between Groups A and C (**a**), Groups B and C (**b**), and Groups A and B (**c**) on the basis of LEfSe analysis (taxa with LDA score ≥ 4, *p* < 0.05).

Microbial contributions to between-group differences among the dietary groups were assessed using the LDA score. The results for the cecum comparisons (A vs. C) are illustrated in Figure 10a; the relative abundance of Coriobacteriales, Coriobacteriia, and Actinobacteriota was highest in the C group, but in the A group, that of *Sphaerochaeta* increased. The main taxa that differed between Groups B and C were the Bacilli, which increased in the B group (Figure 10b). The results from Groups A and B are shown in Figure 10c, where Group B, with the high concentration of C-MEA treatment, promoted the enrichment of *Sphaerochaeta*, *Spirochaetia*, *Spirochaetales*, *Spirochaetaceae*, and *Spirochaetota* but decreased the enrichment of *Clostridium_sp_CAG306* and *Bacilli*.

### 3.5. Functional Predictions Based on the Gut Microbiota of Caged Laying Ducks

**Figure 11:**
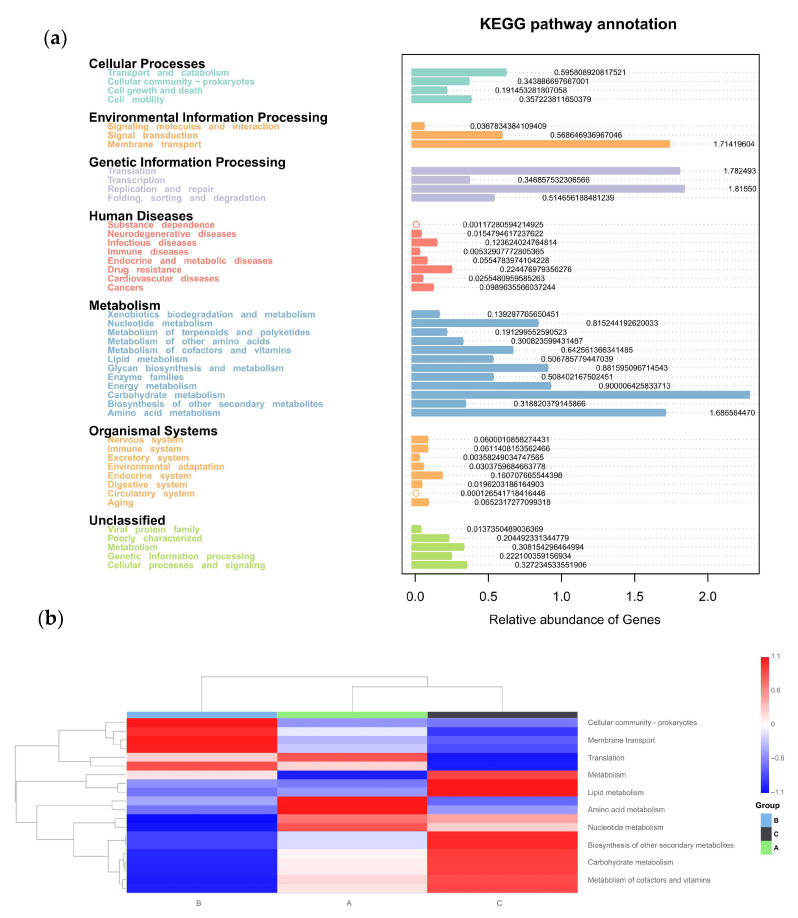
Tax4Fun functional profile of cecal microbiota communities under different dietary groups based on KEGG pathway analysis. (**a**) KEGG pathway prediction based on OTUs among Groups A, B, and C. (**b**) Top 10 KEGG heatmap based on OTUs among Groups A, B, and C.

The physiologic functions of Tax4Fun showed that seven categories of KEGG pathways were classified in all three groups (Figure 11a), including cellular processes, environmental information processing, genetic information processing, human diseases, metabolism, organismal systems, and unclassified. Furthermore, the top 10 KEGGs are listed in Figure 11b. Compared to the control group (Group C), the addition of C-MEA treatment (Groups A and B) inhibited the KEGG pathways of lipid metabolism, biosynthesis of other secondary metabolites, carbohydrate metabolism, and metabolism of cofactors and vitamin, while it promoted the membrane transport pathway.

### 3.6. Correlation Analysis Between the Microbiota, Laying Rate, and Follicular Status of Caged Laying Ducks

**Figure 12:**
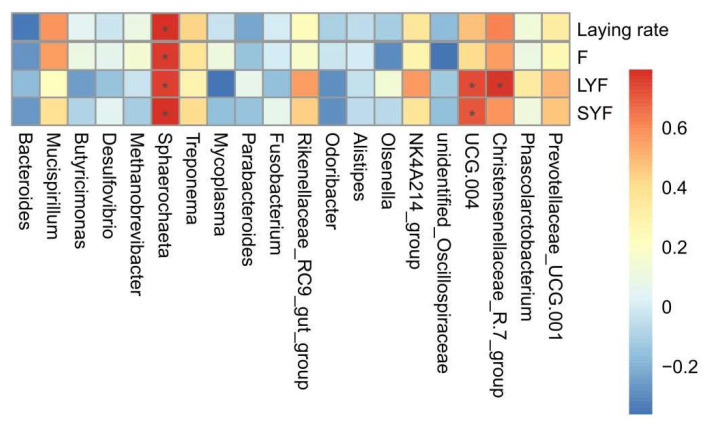
Correlation analysis between the microbiota and variables. Spearman’s correlation analysis between the genus level of the gut microbiota (top 20) and the variables. The color (red to blue) indicates the correlation (positive to negative). * represents a significant correlation strength (*p* < 0.05).

Spearman’s correlation analysis (Figure 12) revealed a positive correlation between *Sphaerochaeta* and all four variables, including laying rate and F, LYF, and SYF numbers (*p* < 0.05). In addition, there were also three positive correlations between *UCG-004* and the LYF numbers (*p* < 0.05), *UCG-004* and the SYF numbers (*p* < 0.05), and *Christensenellaceae-R.7-group* and the SYF numbers (*p* < 0.05).

### 3.7. Correlation Analysis Between the Microbiota and DEGs of Caged Laying Ducks

**Figure 13:**
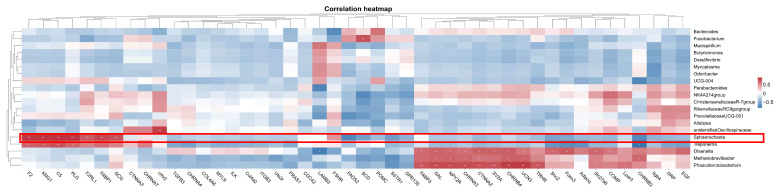
Correlation analysis between the microbiota and key genes. * represents a significant correlation strength (*p* < 0.05), and ** represents an extremely significant correlation strength (*p* < 0.01).

To investigate the potential regulation network between microbiota and mRNA, Spearman’s correlation analysis was performed between the top 20 microbiota genera and 46 candidate key DEGs identified earlier. As shown in Figure 13, a bunch of DEGs showed a positive correlation with microbiota genera; for example, key hub gene *POMC* was positively correlated with *Bacteroides*, and *COMP* was positively correlated with *NK4A214group*. Only two DEGs showed negative correlations: *LAMB3*-*Olsenella* and *CHRNB2*-*Treponema*. The most attention was focused on *Sphaerochaeta*—a genus previously found to be significantly positively correlated with laying rate among different dietary groups. The analysis revealed positive correlations (*p* < 0.01) between *Sphaerochaeta* and seven genes: *F2*, *KNG1*, *C5*, *PLG*, *F2RL1*, *FABP1*, and *GCG*.

## 4. Discussion

Numerous studies have demonstrated that dietary supplementation with components of Chinese herbal medicine or a microecological agent has positive effects on ducks. Chen et al. [21] reported that honeycomb (one kind of traditional Chinese medicine) extracts could improve the fatty acid composition and amino acid content of duck eggs, thus improving the eggs’ flavor. By use of dietary Artemisia argyi powder, the average laying rate, total PUFA contents of egg yolk, and amino acid levels of eggs had all been improved in laying ducks [22]. Incharoen et al. [23] explored the effects of Bacillus toyonensis BCT-7112^T^ on laying ducks; the compound was shown to enhance egg production, gut health, and microbial diversity. With the cross-development of different subjects, such as microecology, fermentation engineering, and pharmacy, a kind of novel microecological product, the Chinese herbal medicine compound microecological agent (C-MEA), has sprung up. This formulation can make good use of both the efficacy of Chinese herbal medicine and the microecological agent through their synergistic actions [24,25]. C-MEA may have the potential to develop into a substitute for antibiotics in poultry production, but there are few studies on C-MEA on laying ducks. Our results indicated that feeding ducks with 8 or 16 g/kg of C-MEA was able to clearly increase both egg production and the number of follicles of caged laying ducks during the 30 days of the formal experiment. The above results indicated that cage rearing induces greater stress in laying ducks, potentially causing irreversible reproductive system damage manifested as ovarian dysfunction and significantly reduced egg production. However, the C-MEA promoted grade follicle development in the ovaries of caged ducks and effectively maintained egg production efficiency compared to the control group. One hypothesis for improved production performance is related to the microbiota–gut–ovary axis, in which intestinal microbiota are exploited to enhance the ovary function of laying poultry to improve laying performance. To investigate the potential regulation mechanism of C-MEA on egg performance, we conducted ovary RNA sequencing and cecal 16S sequencing of caged laying ducks among different dietary groups.

The poultry’s ovary is a crucial reproductive organ that directly influences the egg production trait. RNA sequencing data analysis comparing three dietary inclusion levels of C-MEA in the ovaries of caged laying ducks revealed a total of 16,726 DEGs. To obtain a preliminary understanding of the function of these DEGs, top 30 GO enrichment analysis was carried out, and their functional roles were mainly in egg production, such as the G-protein-coupled receptor pathway, calcium ion binding, and extracellular matrix. To narrow down the potential candidate key genes that may regulate egg production based on the addition of C-MEA, KEGG enrichment analysis was further carried out. Based on the significance levels of KEGG pathways and the literature reviews of the function of these pathways, this study mainly focused on neuroactive ligand–receptor interaction [26], the PPAR signaling pathway [27], ECM–receptor interaction [28,29,30], focal adhesion [31,32,33], the adherens junction [34,35], and the FoxO signaling pathway [36,37], which are involved in egg production performance or follicular development. Therefore, a total of 73 DEGs were enriched from the above six signaling pathways, among which 46 DEGs showed interaction with each other and were defined as candidate key genes in this study, including *POMC*, *FSHR*, *EGF*, *FOXO1*, *FOXO3*, *GCG*, *ITGB3*, *ITGB4*, *F2RL1*, and *FABP1*. The top enriched hub gene was *POMC* (Pro-opiomelanocortin), a polypeptide precursor of several peptide hormones and neuropeptides, which plays an important role in mediating stress, health, survival, and reproduction [38,39]. Liu et al. [40,41] detected the polymorphism of *POMC* in Zhenning yellow chickens and explored the SNP of g.1140C > T that was associated with the E300 egg production strain, while the SNPs of g.958 G > A and g.1817 C > T were significantly associated with E_2_ hormone levels and reproduction traits in chickens. Mu et al. [42] compared the ovarian transcriptome of low- and high-yielding Changshun green-shell laying hens, and *POMC* was found to be significantly expressed in high-yielding groups and identified to be the candidate gene for the improvement in egg production. *POMC* mRNA expression was also demonstrated in the hypothalamus of Shaoxing ducks, which was then indicated to initiate the sexual maturation of ducks [43]. Lei et al. [44] immunized laying chickens with LEPR ECD and found that a decreased egg-laying rate and follicle atresia were accompanied by the upregulation of *POMC*. All these studies indicated that *POMC* was involved in follicle development and egg production. Our study indicated that only feeding ducks with 8 g/kg of C-MEA clearly decreased the expression of *POMC*, which was also accompanied by an increase in F, LYF, and SYF numbers. These findings highlight the complex interplay of key pathways and hub genes in modulating reproductive behaviors and genetic regulation mechanisms underlying caged environments in laying ducks.

The cecum plays a crucial role, as it houses a vast and diverse microbial community essential for fermenting and digesting poultry feed [45]. Consequently, investigating how changes in cecal microbiota regulate growth and development in livestock and poultry is of significant importance; however, research on caged laying ducks remains limited. Therefore, this study aims to further explore the effects of C-MEA on cecal microbiota in caged laying ducks by comparing 16S sequencing data between different dietary groups. The experimental results showed that the estimated values of Group A (Chao1 and Simpson) increased significantly compared to the control group (Group C), indicating that the species richness of the cecal digestive tract was higher. Some research concluded that higher intestinal microbe abundance makes animals better able to cope with environmental disturbances, and the distinct microecological spaces critically impact individual nutrition, immunity, disease resistance, and physiological functions, ultimately influencing egg performance [19,46]. *Bacteroidota* was the dominant phylum in all three groups, which is responsible for the fermentation of complex indigestible polysaccharides through its enzymatic capacity for carbohydrate utilization [47]. We also discovered that at the phylum level, the *Spirochaetota*, *Synergistota*, and *Verrucomicrobiota* abundances significantly increased in caged laying ducks supplemented with C-MEA, while the abundance of *Bacteroidota* and *Actinobacteriota* significantly decreased; at the genus level, the relative abundances of *Sphaerochaeta* and *UCG-004* were significantly increased in Group A. Furthermore, correlation analysis showed that *Sphaerochaeta* was positively correlated with E30 laying rate and F, LYF, and SYF numbers among different dietary groups. The genus *Sphaerochaeta* is composed of chemoorganoheterotrophic anaerobes with fermentative metabolism and related to carbohydrate (pentose, hexose, disaccharides, and soluble starch) metabolism [48]. It has been reported that the alteration in *Sphaerochaeta* might affect the metabolism of laying hens to improve their egg quality [49]. Moreover, the abundance of *Sphaerochaeta* was found to be positively related to the chicken egg-laying rate of broiler breeders [50]. It was speculated that the addition of C-MEA increased the abundance of *Sphaerochaeta* and improved the health of the intestine, which might produce the precursors for the synthesis of hormones related to the egg production of ducks. The specific mechanisms by which C-MEA increases host health via their effects on the microbiota–gut–ovary axis need to be further explored. Correlation analysis between the 46 candidate key DEGs (RNA sequencing) and the top 20 microbiota genera (16S sequencing) from all three different dietary groups showed that *Sphaerochaeta* was extremely positively correlated with *F2*, *KNG1*, *C5*, *PLG*, *F2RL1*, *FABP1*, and *GCG* (*p <* 0.01). Due to the short experimental duration and the absence of hormonal analysis, future research will need to be carried out to verify the mechanisms of interaction between hub genes and microbiota on C-MEA and lay the foundation for improving gut health and reproductive performance in caged laying ducks.

## 5. Conclusions

This study examined the synergistic effect of a C-MEA and confirmed that the effect was most prominent when administering 8 g/kg, significantly increasing the egg-laying rate and F, LYF, and SYF numbers. Combining the RNA-Seq and 16S-Seq results, it can be reasonably deduced that egg production performance can be regulated through the microbiota–gut–ovary axis of caged laying ducks. Notably, the relationship between *Sphaerochaeta* and the hub gene cluster (*F2*, *KNG1*, *C5*, *PLG*, *F2RL1*, *FABP1*, and *GCG*) is the most prominent, which requires further experimental validation to confirm the regulation of the microbiota–gut–ovary axis of C-MEA.

## Data Availability

Data produced in this study are available from the corresponding authors upon reasonable request.

## Supplementary Materials

The following supporting information can be downloaded at https://www.mdpi.com/article/10.3390/vetsci12090808/s1: Table S1 Primer information for qRT-PCR, Table S2 Quality evaluation and analysis of preprocessing RNA-Seq data, and Table S3 Number of reads that passed through each step of paired-end reads assembly and quality control.
