# Supplementary material for: Chinese Herbal Medicine Compound Microecological Agent (C-MEA) Improves Egg Production Performance in Caged Laying Ducks via Microbiota–Gut–Ovary Axis
Source: Vet Sci. 2025 Aug 25;12(9):808. doi: 10.3390/vetsci12090808 (PMC12474147; doi:10.3390/vetsci12090808)
Supplement: Supplementary file 1 [file vetsci-12-00808-s001.zip › Supplementary Table S1.pdf]

Supplementary Table S1. Primer information for qRT-PCR

Gene	Sequence (5′-3′)	Tm (°C)
FSHR	F: AGTGTTGAATGTACTCGCCT<br>R: TGTCTGTTCTGTAAATCTGG	60
FOXO3	F: ACAAACTCCATCCGGCACAA<br>R: AGGGCCGCCTTTTTCTTAGC	59
LHCGR	F: CATTTGACAGCCTTCCTGCT<br>R: CGCAAGTTATCACAGAGCTCCA	60
PGF	F: AAGGAGATCTGGAATCGCAGC<br>R: GACATCTCCACGTAGGGTGC	60
FABP1	F: AGGGCAAGGACATGAAGAGC<br>R: ACGGATTTCAGCCCCTTCAG	60
COL4A2	F: GAGTGCAAGGTCCCAAAGGT<br>R: GGCAATGTCCGGTGGTAGTA	59
EGF	F: ATGCTGGTCCGTCAACACTT<br>R: AGTCTCTCTCGCTTTGCACC	60
β-actin	F: CAGCCATCTTTCTTGGGTAT<br>R: CTGTGATCTCCTTCTGCATCC	60
