# Supplementary material for: Chinese Herbal Medicine Compound Microecological Agent (C-MEA) Improves Egg Production Performance in Caged Laying Ducks via Microbiota–Gut–Ovary Axis
Source: Vet Sci. 2025 Aug 25;12(9):808. doi: 10.3390/vetsci12090808 (PMC12474147; doi:10.3390/vetsci12090808)
Supplement: Supplementary file 1 [file vetsci-12-00808-s001.zip › Supplementary Table S2.pdf]

Supplementary Table S2. Quality evaluation and analysis of preprocessing RNA-Seq data.

sample	raw_reads	raw_bases	clean_reads	clean_bases	Q30	GC_pct
A1	48155756	7.22G	44314252	6.65G	93.35	48.47
A2	47625230	7.14G	45020640	6.75G	94.21	46.66
A3	46330666	6.95G	44597556	6.69G	93.91	46.49
B1	49194710	7.38G	47265796	7.09G	93.70	50.46
B2	46054518	6.91G	43355294	6.5G	93.96	51.48
B3	45252058	6.79G	43497654	6.52G	93.32	49.03
C1	46253908	6.94G	41633694	6.25G	94.08	51.03
C2	45936860	6.89G	43453570	6.52G	93.33	47.10
C3	43482166	6.52G	42813240	6.42G	92.81	47.11
