# Supplementary material for: Chinese Herbal Medicine Compound Microecological Agent (C-MEA) Improves Egg Production Performance in Caged Laying Ducks via Microbiota–Gut–Ovary Axis
Source: Vet Sci. 2025 Aug 25;12(9):808. doi: 10.3390/vetsci12090808 (PMC12474147; doi:10.3390/vetsci12090808)
Supplement: Supplementary file 1 [file vetsci-12-00808-s001.zip › Supplementary Table S3.pdf]

Supplementary Table S3. Number of reads that passed through each step of paired-end reads assembly and quality control.

Sample	RawPE	Combined	Qualified	Nochime	Base(nt)	Avglen(nt)	GC	Q30	ASV
A1	103159	102880	102227	82973	20976809	252.81	53.00%	96.55%	747
A2	103548	103242	102483	86765	21945227	252.93	52.62%	96.47%	805
A3	105867	105578	104977	95131	24060328	252.92	52.13%	96.75%	720
B1	103932	103618	103011	97702	24702217	252.83	52.79%	96.44%	875
B2	102123	101972	101652	97637	24693077	252.91	51.00%	97.35%	574
B3	149850	148490	148008	136027	34175802	251.24	52.33%	97.89%	480
C1	60315	60151	59907	56883	14487380	254.69	51.10%	97.31%	235
C2	140142	139044	138611	125593	31764847	252.92	53.08%	97.86%	1057
C3	64854	64736	64472	60596	15331968	253.02	52.08%	97.21%	583
